# Development of a human malaria-on-a-chip disease model for drug efficacy and off-target toxicity evaluation

**DOI:** 10.1038/s41598-023-35694-4

**Published:** 2023-06-28

**Authors:** Michael J. Rupar, Trevor Sasserath, Ethan Smith, Brandon Comiter, Narasimhan Sriram, Christopher J. Long, Christopher W. McAleer, James J. Hickman

**Affiliations:** Hesperos, Inc., 12501 Research Parkway, Suite 100, Orlando, FL 32826 USA

**Keywords:** Infectious diseases, Malaria, Microfluidics, Biomedical engineering

## Abstract

A functional, multi-organ, serum-free system was developed for the culture of *P. falciparum* in an attempt to establish innovative platforms for therapeutic drug development. It contains 4 human organ constructs including hepatocytes, splenocytes, endothelial cells, as well as recirculating red blood cells which allow for infection with the parasite. Two strains of *P. falciparum* were used: the 3D7 strain, which is sensitive to chloroquine; and the W2 strain, which is resistant to chloroquine. The maintenance of functional cells was successfully demonstrated both in healthy and diseased conditions for 7 days in the recirculating microfluidic model. To demonstrate an effective platform for therapeutic development, systems infected with the 3D7 strain were treated with chloroquine which significantly decreased parasitemia, with recrudescence observed after 5 days. Conversely, when the W2 systems were dosed with chloroquine, parasitemia levels were moderately decreased when compared to the 3D7 model. The system also allows for the concurrent evaluation of off-target toxicity for the anti-malarial treatment in a dose dependent manner which indicates this model could be utilized for therapeutic index determination. The work described here establishes a new approach to the evaluation of anti-malarial therapeutics in a realistic human model with recirculating blood cells for 7 days.

## Introduction

Malaria cases have been on the rise from 2014 to 2020, with reported cases increasing from 217 to 229 million^1^. With this steady increase in reported cases and the emergence of resistant strains over the years, it is incredibly important to develop novel approaches to anti-malarial development to combat disease progression. Malaria, a disease caused by the *Plasmodium* parasite, is transmitted to humans via the female *Anopheles* mosquito^2^. While there are various species of *Plasmodium* (e.g., *malariae, ovale, knowlesi, vivax*), the *falciparum* species is the deadliest and is primarily responsible for many severe malaria cases^3^. Once transmitted to humans, the parasitic life cycle exists in two different stages. During a blood meal, sporozoites are released from the saliva of the mosquito. Sporozoites then travel through the peripheral blood circulation to the liver where they replicate inside of hepatocytes. This process results in the abundant formation of merozoites in a matter of days. Merozoites are then released from ruptured hepatocytes and navigate from the liver to the bloodstream. Merozoites then initiate the asexual, erythrocytic life cycle of the parasite via infection of red blood cells. Here, they undergo rapid asexual replication fueled by the hemoglobin of the host’s red blood cells. Within 48-h, merozoites enter a ring stage, which mature to become trophozoites, and ultimately schizonts. The schizonts will continue to grow and replicate until they burst, releasing more merozoites and repeating the cycle of infection^4,5^.

The World Health Organization has proposed a goal to globally reduce both malaria case incidences and mortalities by 90% by the year 2030. To achieve this goal, it is important to investigate new platforms for how researchers study disease remedies. Animal models, such as the use of non-human primates and humanized mice, have greatly contributed to the understanding of malaria and are currently used for anti-malarial drug discovery. Although the preclinical data obtained from these models have been crucial in the progression of anti-malarial development, they are not without limitations. One reason being that non-human malaria parasites are used in these models. This introduces variability in rate of infection and sensitivity to drug compounds. There have been strides in modeling advancements, such as studies that successfully cultivated *P. falciparum* in immunodeficient SCID mice^6,7^. Aside from the obvious deficits in immune interference, these models also lack the ability to replicate parasite sequestration as observed during interaction with human endothelial cells.

To address these concerns, new advancements are needed to provide additional models to recapitulate human malaria infection for preclinical drug development. Microphysiological systems (MPS), like the multi-organ malaria-on-a-chip model described in this work, offer new approaches to drug discovery using human-based models in a controlled, self-contained, interconnected platform that offers a more cost-effective alternative to animal models^8,9^. For this report we have constructed a functional, human, multi-organ, serum-free, pumpless system for the culture of *P. falciparum.* One of the initial hurdles to the development of multi-organ microphysiological systems is the development of a medium that allows for the sustainability of all organ constructs contained within the system^9^. Previous work has outlined the development of a defined, serum-free base medium system that enables culture of a wide range of cell types up to several months and removes a major variable—serum. The exclusion of serum allows for the compatible inclusion of multiple organ-constructs without fear of immunological reactions. Schaffner et al. described the first serum-free, defined culture system for neuronal systems^10^. This system can satisfactorily support cardiac^11–13^, hippocampal neurons^14^, motoneurons (MNs)^15,16^, sensory neurons^17,18^, muscle^19^, NMJ formation^20^, liver^21^ and endothelial and epithelial cells. Over time this serum-free formulation as well as others has demonstrated satisfactory support an array of organ constructs, including endothelium and liver constructs and multi-organ systems^22^. Throughout the years various researchers have successfully utilized serum replacements in media for cultivation of *P. falciparum*^23–26^.The combination of these approaches enabled the cohesive assembly of primary human hepatocytes, human umbilical vein endothelial cells (HUVECs), recirculating primary human red blood cells (RBCs), as well as the inclusion of primary human splenocytes in a multi-organ system. Organ constructs were selected based on the clinical pathophysiology of the malaria disease conditions. The spleen is vital in clearance for the parasite from infected erythrocytes^27–29^. The spleen also provides both mechanical and immunological defenses against *P. falciparum.* Once infected, RBCs lack deformability and may become trapped within the sinusoids of the spleen. Confined localization of parasites assists antigen presenting cell populations, like dendritic cells and T cells, to elicit an immune response while enabling phagocytic clearance of parasitic material by macrophages.

During the erythrocytic life cycle of the parasite, infected red blood cells (iRBCs) become coated with *Plasmodium falciparum* erythrocyte protein 1 (PfEMP-1)^30–32^. This increased protein expression results in sequestration of iRBCs as PfEMP1 specifically binds to intracellular adhesion molecule 1 (ICAM1), expressed by endothelial cells, and this phenomenon is known as cytoadherence. Due to the lack of human endothelial cells in current in vitro malaria models HUVECs were included in an attempt to study sequestration. As parasite infected cells cytoadhere to endothelium they effectively escape splenic clearance, thereby prolonging parasite proliferation resulting in chronic infection. Cytoadherence of iRBCs to endothelial linings is also observed in placental and cerebral malaria^33–36^. Inclusion of the liver organ construct provides insightful information for the evaluation of drug efficacy in vitro and to determine translation for in vivo relevance^37,38^. As optimal anti-malarial doses are investigated to reduce parasitemia in this disease model, the addition of hepatocytes also helps to evaluate pharmacokinetics and the effects of metabolism in the system^39^. Determination of compound bioavailability and off-target toxicity after hepatocyte metabolism helps to produce a more predictive preclinical model.

The system described above is a prospective new human model of malaria for use in pre-clinical anti-malarial drug discovery that demonstrates the establishment and maintenance of both healthy and disease conditions. Two strains of *P. falciparum*, both a chloroquine sensitive and chloroquine resistant strain, were successfully maintained over an 8-day period in the multi-organ model with no significant decline in parasite or organ construct viability when compared to the healthy system. This development allowed further evaluation of chloroquine treatment for both therapeutic efficacy as well as off-target toxicity, where systems infected with the 3D7 strain showed a significant decrease in parasitemia levels, with recrudescence observed after 5 days. Conversely, when systems infected with the W2 strain were dosed with chloroquine, parasitemia levels were moderately decreased when compared to the 3D7 infected systems. The system also evaluated the off-target toxicity of the anti-malarial treatment in a dose dependent manner and indicated increased toxicity at higher doses of chloroquine. This multi-organ platform establishes a new methodology to evaluate malaria therapeutics in a human system with the ability to evaluate efficacy and safety within the same platform, which enables the determination of therapeutic index in future studies.

## Results

### Multi-organ system design

**Figure 1:**
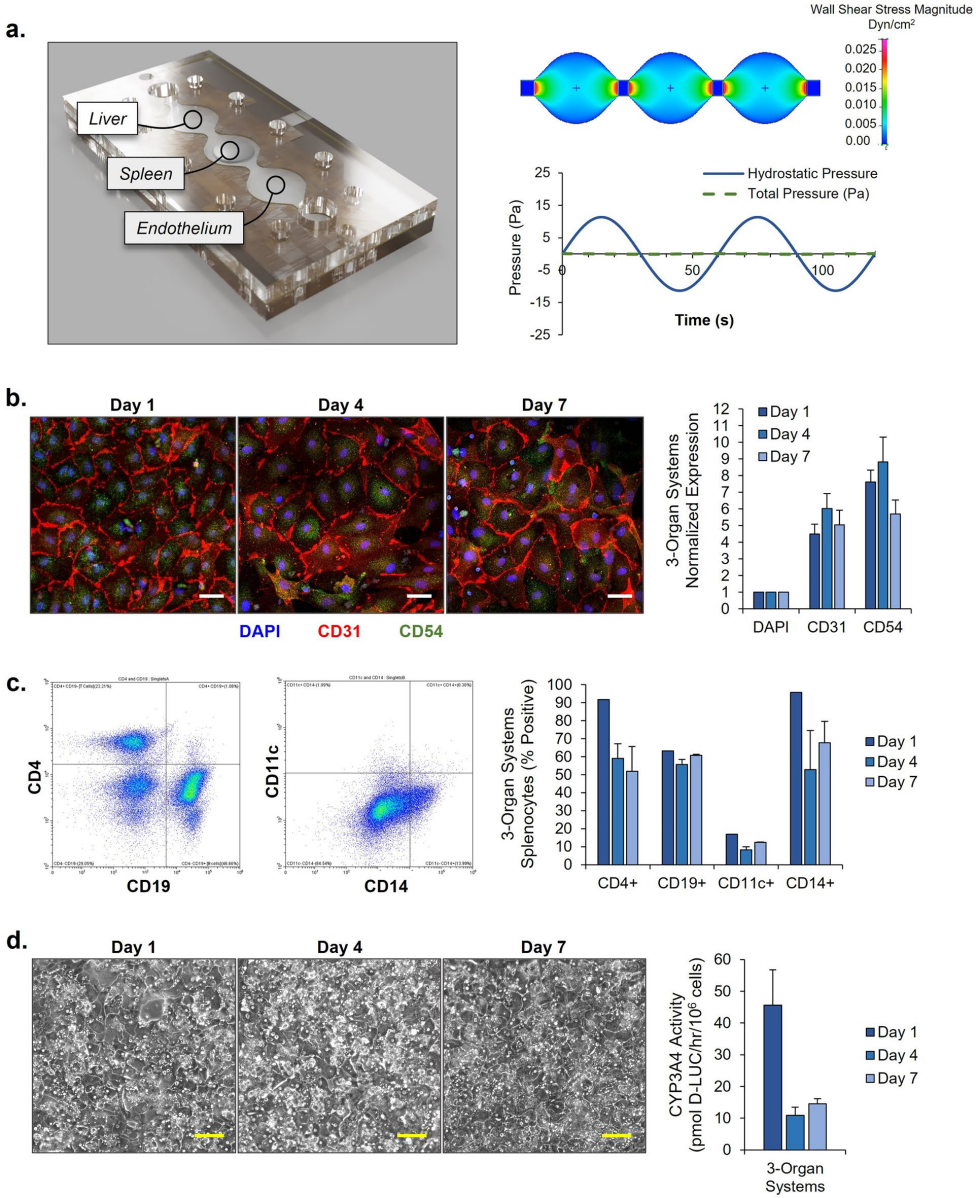
3-Organ human-on-a-chip system for the housing of functional human organ constructs. (**a**) A three-dimensional representation and flow profile analysis of the multi-organ system developed for in-vitro study of *P. falciparum*. (**b**) Endothelium morphology and marker expression profile. Immunocytochemical characterization and area quantification of CD31 and CD54 expression by HUVECs in 3-Organ systems. Expression of CD31 and CD54 were normalized to DAPI nuclear stain. Scale bars = 40 µM (**c**) 3D spleen construct marker expression profile. Gating strategy employed for flow cytometry analysis of typical splenocyte markers. Quantification of viability and characteristic splenocyte markers on coculture at days 1, 4, and 7. Error bars ± SEM. (**d**) Phase image morphology of primary hepatocytes in the multi-organ microfluidic system. Scale bars = 100 µM. Hepatocyte viability as determined by MTT assay. Error bars ± SEM.

For this project, a serum-free, microfluidic device was developed that contained three separate compartments for plating of liver, spleen, and endothelial cells (Fig. 1A). This pumpless system introduces gravity-driven flow to the multi-organ system with continuous sinusoidal rocking. Relevant physiological parameters were established in accordance with previous research^40,41^ to ensure an accurate model of physiological flow and shear stress in the platform. The use of continuous flow allows for interaction amongst organ constructs, even distribution of medium, and systemic delivery of therapeutic compounds. Specifically, for the fabrication of this malaria-on-a-chip disease model, use of microfluidics without pumps also allows for the recirculation of primary human red blood cells. Thus, enabling systemic infection with the erythrocytic, asexual stage of the *P. falciparum* parasite.

### Characterization of cells in the multi-organ system

Cells were initially assembled on chips and characterized without the presence of the *Plasmodium falciparum* in the malaria-on-a-chip platform. This characterization was executed for a 7-day period and allowed for the determination of cell viability, function, and morphology in a healthy control system. Endpoint assays were performed on days 1, 4, and 7. Standard, static monocultures of each cell type were performed in tandem with the microfluidic multi-organ model. No statistically significant differences were observed (results not shown). The endothelium constructs (Fig. 1B) were imaged at each endpoint via phase imaging. This showed that the HUVECs continued to form a homogeneous monolayer while exposed to microfluidic conditions. These cells were also stained for DAPI, CD31 (PECAM-1), and CD54 (ICAM-1) for imaging via immunocytochemistry. Red blood cells infected with the *P. falciparum* parasite express PfEMP1 which binds to the endothelial cell receptor ICAM1. The presence of CD54 allows for the cytoadhesion of infected red blood cells to be potentially studied in the platform. The spleen constructs (Fig. 1C) were also imaged via phase imaging while embedded in the hydrogel at each endpoint. The hydrogel was dissolved and the splenocytes, consisting of T cells, B cells, macrophages, and dendritic cells, were fixed and stained for flow cytometry. Cell markers for each of these cell populations, such as CD4, CD19, CD11c, and CD14 were all observed. The persistence of these immune cell populations for the spleen construct allows for future studies to be performed to observe splenic clearance of infected red blood cells in these systems. For the liver construct (Fig. 1D), primary hepatocytes were imaged via phase imaging at each endpoint. The expected cobblestone morphology of the hepatocytes was maintained over the course of the 7 days. In addition to standard morphology of the cells, MTT assays were performed and found no statistically significant decrease in cell viability over the 7-day period (results not shown).

### Plasmodium falciparum life cycle in the system

**Figure 2:**
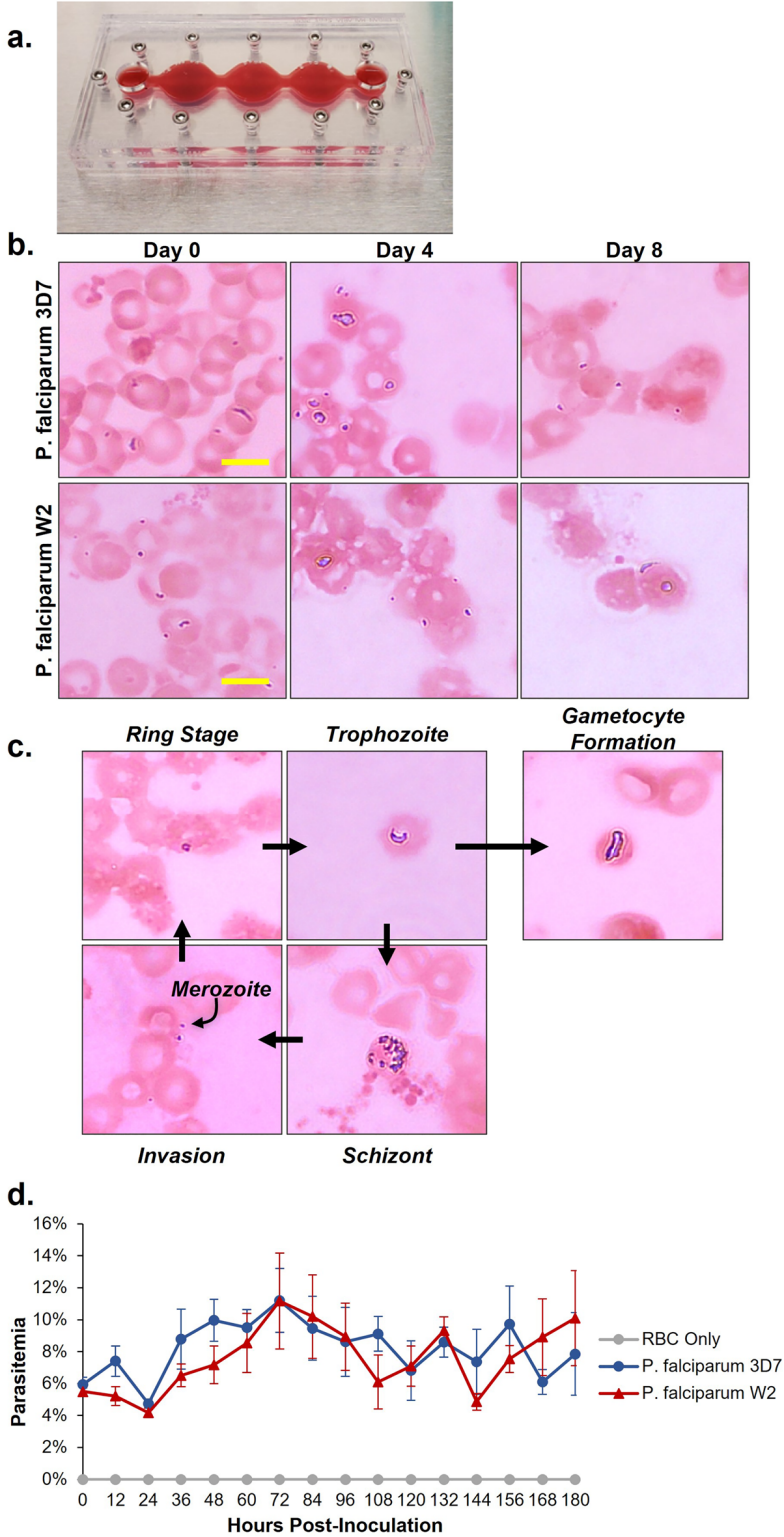
Morphological progression of *P. falciparum* in the 4-organ malaria-on-a-chip system. (**a**) Multi-organ housing after addition of 3% primary human red blood cells. (**b**) On the day of system assembly, infected erythrocytes were drawn from continuous cultures and each system was inoculated at 5–7% parasitemia. Both 3D7 and W2 strains of *P. falciparum* proliferated over an 8-day time frame in the systems at 2–6% hematocrit, as determined by thin blood smear and Giemsa stain. Scale bars = 5 µm. (**c**) Parasites at all stages of the erythrocytic life cycle were observed after four days of culture in the multi-organ systems, including parasites at stage III of gametocyte formation. (**d**) Parasitemia was determined every 12 h via thin film smear with Giemsa stain. Both strains oscillated between 4–14% parasitemia when cocultured in the 4-organ systems. Data points represent mean ± SEM.

Two strains of *Plasmodium falciparum* were used for this project. The 3D7 strain and the W2 strain, which are chloroquine sensitive and chloroquine resistant, respectively. Both strains were continuously cultured in separate T-25 flasks and kept in hypoxic conditions. Before dosing, multi-organ systems were inoculated with the parasites to determine the viability of *P. falciparum* under flow. For initial characterization of parasite viability in the systems, functional cells were excluded, and only red blood cells were cultured (Fig. 2A). Platforms were inoculated with either the 3D7 strain, W2 strain, or uninfected red blood cells as a control. All conditions were cultured under flow for 8 days. Medium changes were performed daily, and every 2 days fresh, uninfected red blood cells were added to the systems to maintain the hematocrit as well as bolster proliferation of the parasites. For each strain, parasites were observed over the entire 8 days (Fig. 2B) and various stages of the parasites’ lifecycle were observed in the multi-organ platform (Fig. 2C). As the parasite transitions from the liver infection stage to the erythrocytic cycle, merozoites begin infiltrating RBCs. As the merozoites invade the RBCs they develop into the ring stage, which matures into a trophozoite phenotype and then ultimately are maintained as schizonts. As the schizonts grow they rupture and cause the cell to lyse, releasing more merozoites for further infection of red blood cells. In some instances, the ring stage can lead to gametocyte formation, transitioning from an asexual stage to the sexual stage of the parasite lifecycle. All these stages were observed throughout the 8 days in the platform. Every 12 h a 10 µL sample was collected from the systems and used to perform a thin blood smear and giemsa staining of the blood smears was used to determine the parasitemia levels in each system (Fig. 2D).

### Viability of functional cells in the systems

**Figure 3:**
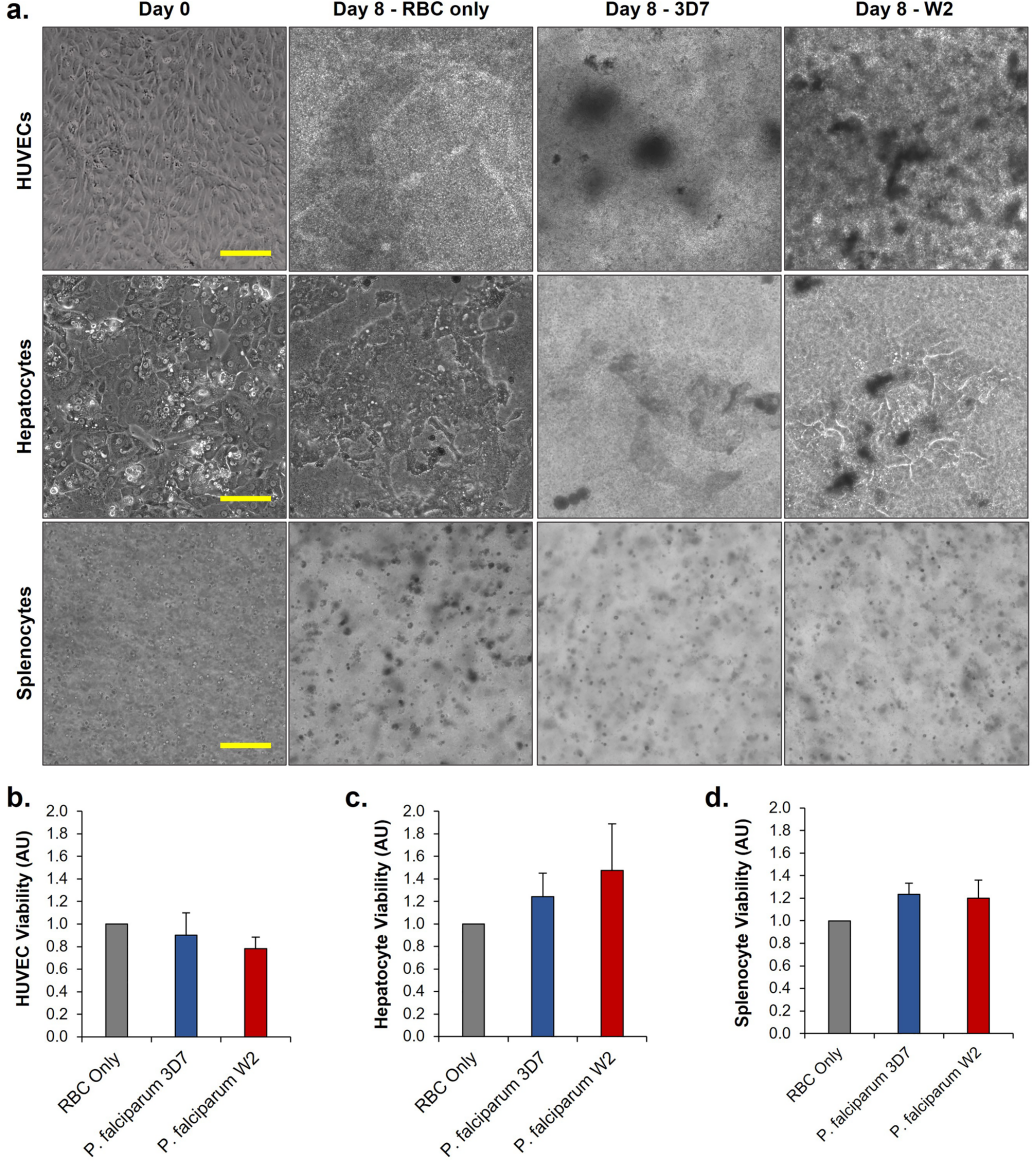
(**a**) Morphologies of endothelium, spleen, and liver organ constructs after 8 days of coculture in 4-organ systems with recirculating uninfected or infected erythrocytes (RBCs). Across all conditions, the presence of concentration of erythrocytes required for parasite growth made imaging these organ modules difficult. However, based on apparent morphological differences between organs in uninfected (RBC only) and infected (3D7, W2) systems, the presence of *P. falciparum* in culture appears to significantly affect the morphologies of these cells. (**b**–**d**) Viability of HUVECs, primary hepatocytes, and primary splenocytes. No statistically significant differences were observed; n = 12.

The single-organ blood model was then integrated with the 3-organ system to develop the platform. Systems either contained red blood cells infected with the 3D7 strain, the W2 strain, or uninfected red blood cells for a control and cultured for 8 days. Phase imaging was used to capture the functional cells on day 0 immediately before assembly into the systems, as well as on day 8 immediately after disassembly of the systems (Fig. 3A). Due to the coculture with the red blood cells, images of each of the organs became obstructed in the platforms. However, some morphological changes could be observed suggesting that infection with *P. falciparum* could disrupt cell viability. The viability of the HUVECs and the splenocytes were analyzed using Alamar blue metabolic reduction assay and hepatocyte viability was analyzed using the MTT assay (Fig. 3B-D). Both viabilities for the infected systems were normalized to the uninfected control systems. No statistical difference was observed between either system.

### Adsorption of chloroquine in the 4-organ systems

**Figure 4:**
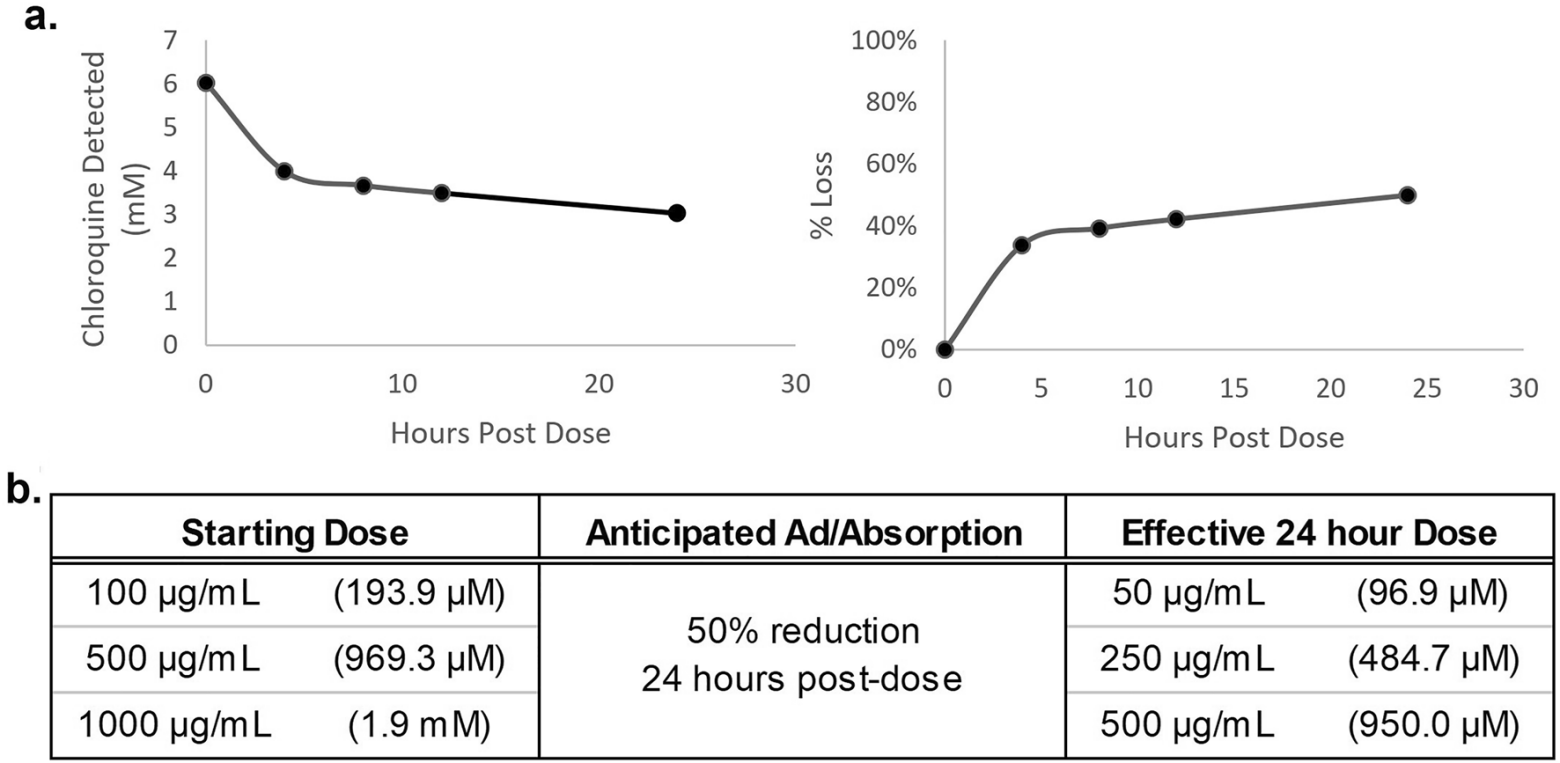
Chloroquine ad/absorption in acellular malaria-on-a-chip systems. (**a**) Acellular systems were dosed with a solution of 6 mM chloroquine and samples were drawn at 0, 4, 8, and 24 h. Drug content in each sample was quantified by high performance liquid chromatography mass spectroscopy (HPLC–MS). Recorded concentrations were fit to a first order asymptotic function for the starting concentration, equilibrium concentration, and the rate constant. (**b**) Doses utilized in the 4-organ systems (left column) compared to their effective average dose after a 24-h exposure to chloroquine (right column).

Before the addition of chloroquine to the malaria-on-a-chip systems, the binding affinity of chloroquine phosphate within the housings was determined. The PDMS and the acrylic used in the platform is hydrophobic and as a result, when evaluating therapeutic compounds such as chloroquine phosphate, they may bind to the interior of the systems. To determine the degree to which the compound binds to the system interior, acellular systems were treated with 1000 µg/mL (6 mM) of chloroquine phosphate and aliquots of media were collected at 0-, 4-, 8-, 12-, 24-h timepoints. Previously studies^39,42^ determined the time-dependent behavior of compounds within these systems and it was found that while each compound differs in the extent of ad/absorption, the profile shapes are repeatable, and thus the overall profile can be estimated from later time points prior to medium changes. Thus, it was determined that the average exposure over the initial 24-h period was approximately 50% of the nominal starting dose, indicating that chloroquine has a moderate binding affinity to the system interior. A linear approximation of the ad/absorption for this concentration was extrapolated and applied to all concentrations to inform the relative behavior of the compound within the systems. Through these findings it was approximated that the 100 µg/mL, 500 µg/mL, and 1000 µg/mL administered doses were equivalent to 50 µg/mL, 250 µg/mL, and 500 µg/mL after 24 h of exposure (Fig. 4). Furthermore, for this study chloroquine was delivered as a single bolus dose with no additional chloroquine added during medium changes. Considering the half life of chloroquine phosphate far surpasses the time the systems were maintained in culture; it was expected that the concentration would decrease by 30% each day in vitro due to the daily 30% medium change.

### Hematocrit and parasitemia in the malaria-on-a-chip systems with chloroquine treatment

**Figure 5:**
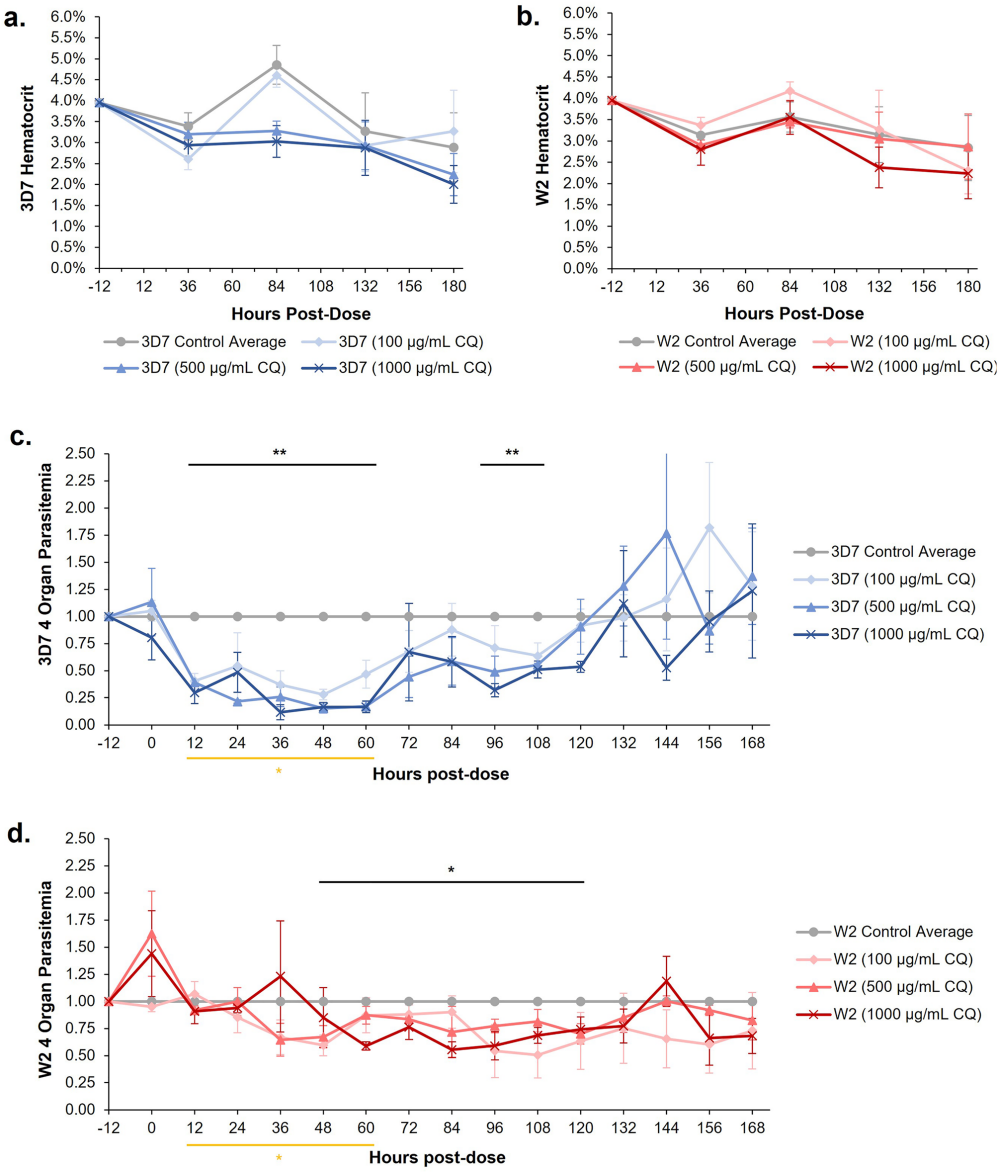
Effects of chloroquine dosing on hematocrit and parasitemia of cells in 4-organ systems. (**a**, **b**) Hematocrit in both 3D7 and W2 4-organ systems was maintained between 2 and 5% with the addition of fresh, uninfected erythrocytes every 48 h. (**c**) Similar to the effects observed in the single-organ dosing experiments, acute exposure of 3D7 systems to chloroquine resulted in a decrease in parasitemia by 12 h post-dose. This decrease in parasitemia appears to be semi-dose dependent with complete recrudescence in these systems by the 132-h mark. (**d**) Parasitemia in W2 4-organ systems did not decrease below controls until 48 h post-dose and achieved complete recrudescence by hour 132. Black lines indicate grouped parasitemia that falls below control baseline. The yellow line indicates that for hours 12 to 60, 3D7 parasitemia is statistically distinct from W2 parasitemia with a *P* ≤ 0.05. Data was normalized to control at each timepoint, data points are mean ± SEM. n = 12; *P* values = *≤ 0.05, **≤ 0.01.

The chloroquine sensitive (containing the *P. falciparum* strain 3D7) and the chloroquine resistant (containing the *P. falciparum* strain W2) systems were acutely dosed with either 100 µg/mL, 500 µg/mL, or 1000 µg/mL of chloroquine immediately after the organ constructs were assembled into the systems. A no dose control was also maintained for each system set. As previously performed in earlier experiments, all conditions were cultured under flow for 8 days with partial medium changes daily. Every 2 days fresh, uninfected RBCs were added to the systems, this allowed for the hematocrit to be maintained throughout the 8 days (Fig. 5A,B). Parasitemia was determined every 12 h and each data point was normalized to the no dose control. An immediate decline in parasitemia levels was observed for the treated chloroquine sensitive systems (Fig. 5C). From hours 12 to 60, parasitemia levels were significantly lower than that of the no dose controls. However, the decrease in parasitemia levels were not maintained, with a slight recovery observed around hour 72 and a complete recrudescence by hour 132. In the treated chloroquine resistant systems (Fig. 5D), while a decline in parasitemia was observed it was not as significant as the decline observed in the chloroquine sensitive systems. The parasitemia levels were significantly less than the no dose control from hours 48 to 120 and returned to baseline after 132 h. However, the decrease was not as drastic as the decrease observed in the sensitive system. It is worth noting that during the 12-to-60-h period, the parasitemia levels of the treated chloroquine sensitive systems were significantly less than that of the treated chloroquine resistant systems.

### Viability of functional cells in the malaria-on-a-chip systems with chloroquine treatment

**Figure 6:**
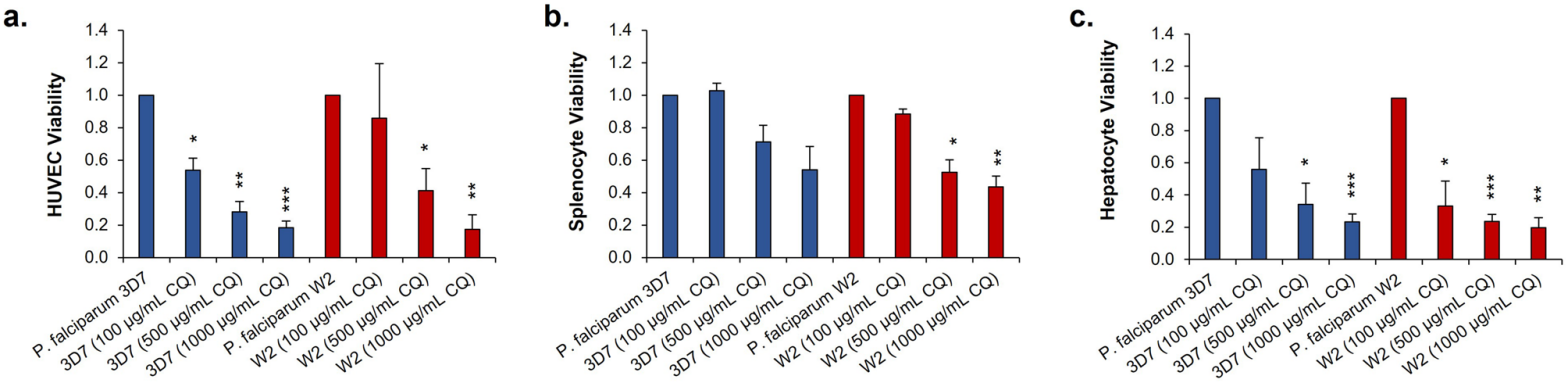
Acute chloroquine exposure negatively impacts the viability of functional cells in a dose dependent manner. (**a**–**c**) Viability of HUVECs, splenocytes, and hepatocytes was negatively impacted by increasing concentrations of chloroquine. Data points are mean ± SEM. n = 12; *P* values = *≤ 0.05, **≤ 0.01, ***≤ 0.001.

Immediately following the disassembly of each system, cell viability was assessed. HUVEC viability was determined via an Alamar blue metabolic reduction assay (Fig. 6A). For both systems, a statistically significantly dose dependent decrease in cell viability was observed, excluding the 100 µg/mL condition of the chloroquine resistant system. The same assay was also performed to determine the viability of the splenocytes (Fig. 6B). Again, a decrease in cell viability was observed in a seemingly dose dependent manner. However, only a statistically significant decrease was only observed for the higher concentrations in the chloroquine resistant systems. As for the hepatocytes, their viability was determined via the MTT assay (Fig. 6C). Once again, a statistically significant decrease in the viability of the hepatocytes with each increase in chloroquine concentration, excluding the 100 µg/mL dosed system in the chloroquine sensitive experiments. All the cell viabilities were normalized to their respective no dose control systems.

## Discussion

The results reported here demonstrate that a 3-organ system was successfully integrated with a single-organ human model of *P. falciparum* infection to fabricate a phenotypic 4-organ malaria disease model. Simultaneously, this model helped to determine any off-target effects of chloroquine on the functionality and viability of the organ constructs contained within the 4-organ platform. This now establishes the ability to determine therapeutic index for antimalarial therapeutics in a similar fashion to our work in determining efficacy and off-target toxicity for chemotherapeutics^42^.

To properly optimize and characterize the *P. falciparum* culture for the development of this disease model, each strain of the parasite was cultured individually and monitored daily. Historically, *P. falciparum* has been cultured in hypoxic conditions (defined as 5% O_2_, 5% CO_2_, and 90% N_2_) in RPMI Complete base medium (RPMI), with daily medium changes and routine addition of fresh RBCs^43^. This traditional methodology was implemented for the expansion of the parasite cultures, allowing maintenance of continuous culture of each strain over the course of experimentation (127 days). Over this time, the continuous cultures were routinely sub-cultured for the purpose of continuous parasite maintenance, cryopreservation for laboratory stocks, or for use in experimental conditions.

Before the addition of parasites to the 3-organ model, a control was performed to affirm that the parasites could be cultured in the same conditions as the functional human cells in the platform. These organ constructs were characterized in normoxic conditions in a serum-free medium formulation, which was labeled “Multi-Organ System Medium” (MOSM). Thus, systems with the same dimensions and flow parameters as the 3-organ systems were used for the development and optimization of a single-organ human model of malaria infection. Systems were maintained in normoxic conditions (defined as 17% O_2_, 5% CO_2_, and 78% N_2_) and both strains were cultured in both the RPMI and MOSM mediums. Daily medium draws were performed over a 14-day period to monitor both the hematocrit and the parasitemia levels. Hematocrit ranges were successfully maintained in all conditions between 2–5% by addition of fresh RBCs to the systems every 72 h. RBCs also maintained similar morphologies as RBCs observed in the traditional continuous culture conditions. Similar parasite proliferation was observed in all conditions, so the inoculation of the 3-organ platform for the development of the phenotypic disease model was initiated.

Individual cultures of each cell type were maintained prior to the assembly of the 4-organ disease model. Primary human hepatocytes, HUVECS, and primary human splenocytes were thawed and cultured 7, 3, and 0 days prior to assembly. This allowed for the cells to achieve functional maturity before assembly into the multi-organ platform containing MOSM under gravity driven flow. Once assembled, either healthy or infected blood was added at a hematocrit of 4%. For the infected systems, unsynchronized cultures of each of the *P. falciparum* strains were added to achieve a parasitemia of approximately 5%. Asynchronous cultures have previously been employed for in vitro drug studies and are also a clinically relevant model as patients commonly present with asynchronous infections^26,44,45^. The parasite density was selected to model hyperparasitemia. Hyperparasitemia in malaria is a condition where greater than 4% of RBCs are infected with parasites. Patients presenting with hyperparasitemia have a proclivity to develop severe malaria and may present resistance to antimalarial treatments^46^. 30% medium changes were performed daily and done so as to not remove any blood from the system. Over the course of 8 days, techniques were performed as previously described to determine parasitemia, hematocrit, and cell viability. These assays confirmed that there were no significant differences in the viability of systems containing uninfected RBCs when compared to the systems containing iRBCs with either strain of *P. falciparum*. Interestingly, while not significant, there was a decline in HUVEC viability that was not observed in the other organ constructs. This may be due to the lack of a collagen topcoat on this organ construct, whereas both the hepatocytes and splenocytes are over-coated with collagen. For this study the sequestration of iRBCs was of interest and the application of a collagen topcoat would prevent direct interaction of the parasitized RBCs with the endothelial cells. This results in a baseline increase in shear stress on the HUVECS. Additionally, the binding of iRBCs to the HUVECs would contribute further to the shear stress. Regardless, it was concluded that infection with these parasite strains is not enough to be significantly detrimental to organ viability for the control. So, any deviations in organ viability observed with the chloroquine dosing experiments should be a direct result of drug exposure.

In previous experiments, single-organ cultures of RBCs and iRBCs were used to determine optimal chloroquine doses for the 4-organ disease model. A low dose group (concentrations of 5 µg/mL, 50 µg/mL, or 100 µg/mL) and a high dose group (concentrations of 500 µg/mL or 1000 µg/mL) were delivered to each single-organ culture (Supplementary Fig. 2). Routine medium collections were performed as described previously. Although dose dependent decreases in parasitemia was observed, it was also worth noting that hematocrit levels dropped drastically. Post treatment, the expected vibrant red color of the systems altered and became a turbid, russet color. When the cells were imaged under a microscope it became clear that chloroquine dosing adversely affected the erythrocyte health.

During infection with *P. falciparum*, the parasite consumes hemoglobin found in RBCs. This results in a toxic byproduct known as heme. Parasites can sequester heme as hemozoin to prevent degradation of parasitic membranes^47–50^. Chloroquine treatment functions to prevent the sequestering of heme, causing a toxic buildup of heme which results in parasite death. Increases in oxidative stress then lead to degradation of cell membranes^51,52^. We feel that due to increased exposure of chloroquine, the RBCs were unable to clear the toxic levels of heme in the system, resulting in hemolytic effects. Consequently, decreased RBC counts and increased membrane fragility was observed. As a result of this, we believe that the turbidity of the medium may be the result of free heme released from ruptured RBCs. To maintain the desired hematocrit range in the systems during acute chloroquine dosing, addition of fresh RBCs was adjusted to 48 h.

Systems were inoculated on the day of assembly (Day 0) with iRBCs drawn from continuous cultures of the 3D7 and W2 strains of *P. falciparum* at approximately 5% parasitemia and 4% hematocrit. Twelve hours after inoculation (Day 1) systems were acutely dosed with chloroquine at either 100 µg/mL, 500 µg/mL, or 1000 µg/mL. As previously performed, 30% media changes were conducted daily, parasitemia was quantified every 12 h via thin blood smear and giemsa stain, and fresh RBCs were added every 48 h. Systems were maintained for 8 days total (7 days post-dose), at which point they were disassembled, and endpoint viability assays were conducted on the HUVECs, hepatocytes, and splenocytes.

Additionally, a dose dependent decrease in functional cell viability was observed. Although unconfirmed, we believe this to be a result of the potential buildup of free heme within the system. Clinically, multiple doses of chloroquine phosphate are delivered within the first 48 h to clear the parasite. However, recrudescence may still occur following treatment and this process over time allows for the emergence of treatment resistance^53,54^. In this proof-of-concept platform for the study of malaria in a MPS model, the objective was to administer supraphysiological doses that would eliminate nearly 90% of the chloroquine sensitive strain. The hope was that delivering a single bolus dose would clear the parasite and still allow for monitoring of recrudescence within the system. The knowledge that the parasite can exist within the system, be cleared through the administered treatment, and return to physiological replication establishes the basic platform for the development of future therapeutics that lack chloroquine disadvantages. To accomplish this, high concentrations of chloroquine were delivered which resulted in a dose-dependent decline in the health of the functional cells, as one may expect. Fortunately, the desired outcome of drastic decreases in parasitemia levels of the chloroquine sensitive systems were obtained even with the lowest acute dose of chloroquine. If we compare each dose’s capacity to reduce a system’s parasitemia to its corresponding off-target toxicity in each organ, we believe that this model can effectively be utilized in ongoing experiments to identify the efficacy and off target toxicity, or therapeutic index, of novel compounds to treat recirculatory erythrocytic parasites. Further, previous work has shown the ability to evaluate effects and off-target toxicity for the metabolites as well as develop PK/PD models of the in-vitro human-based system to predict in-vivo PK/PD effects^39^. Development of an effective assay for observing cytoadherence was also explored and some preliminary data for optical inferences could be made from images obtained via phase and immunocytochemistry (ICC) imaging (Supplementary Fig. 3). Increased clumping of RBCs could be seen in HUVEC coverslips collected from the *P. falciparum* systems. As coverslips were stained for ICC RBCs were no longer visible, suggesting that RBC adhesion may have potentially been disrupted due to excessive washes necessary for fixation and antibody staining. However, increased florescence of CD54 (ICAM1) can be observed in coverslips from *P. falciparum* infected systems when compared to those systems containing only healthy, uninfected RBCs.

## Conclusion

In this report we have demonstrated the development of a novel approach to studying the malaria causing parasite, *Plasmodium falciparum.* With our serum-free multi-organ system, containing liver, spleen, and endothelium organ constructs, a new platform was developed to analyze the disease mechanisms of malaria as well as provide an effective and cost-efficient approach to the evaluation of anti-malarial therapeutic compounds. The organs currently used in the system were chosen to efficaciously observe disease mechanisms such as cytoadherence of infected red blood cells to the endothelium of blood vessels, immune organ responses to parasite infection such as splenic clearance of infected cells, as well as assist in anti-malarial drug discovery by understanding the metabolism of therapeutics by the liver. While further development is still necessary, we believe that this creates a precedent for future progress in the field of malaria research. This allows for the study of the parasite interaction with human cells and to study any foreseeable off-target effects of novel therapeutic compounds, all without introducing any confounding effects of serum in the media.

## Materials and methods

### System fabrication and assembly

System design and fabrication has been previously described for several multi-organ systems^42^. This specific model contains 3 separate housing chambers for liver, spleen, and endothelium; all of which were connected to allow for the circulation of blood and medium throughout the system. Housing consisted of two 6 mm thick clear cast acrylic sheets which formed the top and the bottom halves of the system. These were separated by four 0.5 mm thick poly (dimethyl siloxane) (PDMS) elastomer sheet layers (Supplementary Fig. 4). The acrylic housing and PDMS gaskets were laser cut using a Universal Laser Systems Versa laser PLS 75W laser cutter. The laser cutting of these materials created a defined location for the placement of each organ chip as well as the designation of microfluidic pathways and chambers throughout the system. Prior to system assembly, acrylic housings and PDMS gaskets were soaked in 70% isopropyl alcohol (IPA). Two gaskets were then aligned onto each of the top and the bottom housings and allowed to air dry in a sterile workspace. Before assembly, the housing halves were passivated with 1 × Phosphate Buffered Saline (PBS) with 30 mg/ml of Bovine Serum Albumin (BSA). Passivation of the housing components help to prevent the introduction of air bubbles in the systems, as well as prevent any cells from adhering to the system surfaces. The passivation solution was left on the housings overnight at room temperature. Immediately before assembly, the passivation solution was aspirated, and Multi-Organ Serum-free Media (MOSM) was added in a bubble to the bottom housing unit. All individual organ coverslips were then added to their respective chambers, and top housing units were carefully aligned with the bottom units. The units were then screwed together using a torque screwdriver set to 40in-oz. Systems were incubated at 37 °C and placed on a rocking platform shaker with a defined rocking profile^55^. This allowed for the introduction of gravity-driven, physiological flow, resulting in recirculation of medium through all 3 chambers of the system with defined, minimal levels of shear stress.

### Cell culture

#### Primary human hepatocyte culture

Primary human hepatocytes were obtained from Nova Biosis (Lot #BEI). 7 days prior to system assembly, hepatocytes were thawed and plated. Rat tail collagen (Thermo Scientific cat #A1048301) was used to coat coverslips at a concentration of 60 µg/ml at a seeding density of 250,000 cells/coverslip and maintained in vendor specific medium. One day prior to system assembly, a hydrogel coating was applied to the hepatocyte coverslips. This was performed by aspirating the media from each well, applying 90 µL of hydrogel solution (775 µL 1 × DPBS, 225 µL 3 mg/ml collagen, and 1N NaOH), and incubating the coverslips for 1 h at 37 °C. After incubation, 1 mL of medium was then added back to each coverslip.

#### Human umbilical vein endothelial cell culture

HUVECs were obtained from Lonza (Lot #0000636514). 3 days prior to system assembly HUVECS were thawed and plated. Rat tail collagen (Thermo Scientific cat #A1048301) was used to coat coverslips at a concentration of 500 µg/ml, with a seeding density of 4,500 cells/coverslip and maintained in vendor specific medium.

#### Primary human splenocyte culture

Primary human splenocytes were obtained from BioIVT (Lot # BRH1392583). On the day of system assembly, splenocytes were thawed and plated onto coverslips in a rat tail collagen (Thermo Scientific cat #A1048301) hydrogel coat (2 mg/ml) with a seeding density of 2.0 × 10^6^ cells/coverslip. Cells were incubated in the hydrogel for 1 h at 37 °C. After incubation cells were maintained in vendor specific medium until time of assembly.

#### Plasmodium falciparum culture

Two strains of *P. falciparum* were obtained from BEI Resources; 3D7 is a chloroquine sensitive strain and W2 is a chloroquine resistant strain. Samples were thawed and cultured according to manufacturer’s protocols. Continuous culture of the parasites was then performed as previously described^43^. In brief, parasites were cultured in a T25 tissue culture flask and full medium changes were performed daily with serum free RPMI Complete medium prepared in house (RPMI 1640 [Thermo Scientific cat #23400062], 0.4% Albumax I [Thermo Scientific cat #11020021], 0.21% NaHCO_3_ [Sigma cat #S5761-500G], and 0.0025% Hypoxanthine [Sigma cat #H9377-5G]). The freshly added medium was maintained in a tri-gas atmosphere (5% O_2_, 5% CO_2_, and 90% N_2_). Flasks were stored in an in-house constructed hypoxia chamber to maintain hypoxic conditions (Supplementary Fig. 1). This hypoxia chamber is a sealed chamber constructed from PMMA, with custom hinges and a magnetic sealing lid. The chamber was equipped with oxygen (O_2_) and carbon dioxide (CO_2_) sensors that monitored the environmental gas concentrations in the chamber. A microcontroller (teensy 3.2) was programmed to detect the O_2_ and CO_2_ concentrations and provide feedback control to solenoid valves controlling the gas tanks via a PID algorithm. The microcontroller determined the appropriate duty cycle for each gas cylinder using two proportional controllers configured in a cascade using the Cohen-Coon tuning method. A separate O_2_ sensor (ProOx-110, BioSpherix, Canada) was placed in the incubator to acquire the environmental O_2_ concentration to adjust and monitor for leakage. The closed loop system was programmed to ensure that the average gas concentration was maintained within ± 0.2% of the setpoint concentration.

3D7 cultures were always handled fist, and the workstation was sanitized with 70% IPA before working with the W2 strain to help prevent any potential contamination of the chloroquine sensitive strain. Type O^+^, pooled donor whole blood, stored in CPDA-1 anticoagulant, was obtained from BioChemed every 21 days. Fresh, uninfected red blood cells (RBCs) were isolated from this whole blood every 3 days and added to the parasite culture. An RBC count was performed daily to ensure that a hematocrit of 3% was maintained in the culture. The continuous culture of parasites was maintained for 127 days. These cultures were either sub-cultured to maintain parasite viability in continuous cultures or sub-cultured to infect the platform. Additionally, samples were collected for cryopreservation^56^ to generate laboratory stocks. When added to the systems for experimentation, parasitemia of the culture was determined and added to the systems at 5% parasitemia with a hematocrit of 3%.

#### Multi-organ culture in 4-organ systems

Cells were maintained in the systems with MOSM for a period of 8 days. The total volume of medium within the system was 2mL and 30% medium changes were performed daily. Fresh, uninfected RBCs were added to the systems every 2 days to maintain a hematocrit of 3%. Samples were collected every 12 h post dosing to determine parasitemia levels.

#### Drug preparation and addition to systems

Chloroquine phosphate (Sigma, PHR1258) was added directly to the multi-organ systems on the day of assembly. The compound was dissolved directly into MOSM to create a starting stock of 4 mg/ml. This was then diluted to 3 concentrations of 100 µg/mL, 500 µg/mL, and 1000 µg/mL for dosing of the systems. The drug was administered to the systems on Day 0 immediately after assembly. Approximately a third of the medium (0.6 mL from 2.0 mL) was removed from the systems and replaced with an equal volume of the drug solution.

Preliminary experiments were conducted to determine chloroquine concentrations for dosing in systems containing only blood infected with the 3D7 strain (Supplemental Fig. 1). Initial concentrations included 50 ng/mL, 150 ng/mL, 500 ng/mL, 5 µg/mL, 50 µg/mL, 100 µg/mL, 500 µg/mL, and 1000 µg/mL. Parasitemia was monitored every 12 h for 7 days to determine kill curves. Dose concentrations were selected if a 90% reduction in parasitemia was observed when compared to untreated controls and an observable recrudescence post-dose could be achieved. For the 50 ng/mL, 150 ng/mL, and 500 ng/mL treatments, parasitemia failed to drop below 60% and a complete recrudescence was observed within 24-h. For the 5 µg/mL and 50 µg/mL concentrations, parasitemia was reduced by up to 70% by day 3 and in both a complete recrudescence was observed by day 7. The only doses that achieved a 90% reduction in parasitemia and still achieved recrudescence were the 100 µg/mL, 500 µg/mL, and 1000 µg/mL treatments.

### Giemsa stain

Parasitemia was determined via Giemsa staining. A thin blood smear was prepared using 10 µL of blood obtained from the culture and added to a glass slide. The blood was then smeared across the slide and allowed to air dry. The smear was submerged in a Coplin jar containing absolute methanol (Sigma, cat#322415-4X2L) for 5 min, removed, and then again allowed to air dry. The smear was then added to a second Coplin jar containing freshly prepared, filtered working Giemsa stain (1 mL Giemsa stain:49 mL DI water) (EMD Millipore, cat #R03055-74) for 30 min. After removal from the stain, slides were rinsed for 20 s with DI water and allowed to air dry before observing under a microscope.

### Phase contrast cell imaging

Hepatocyte, splenocyte, and HUVEC morphology was observed via phase imaging prior to assembly and immediately after disassembly in the multi-organ systems (day 0 and day 8 respectively). Images were not obtained during experimentation due to the RBCs obstructing the imaging of the cells. Phase imaging was obtained using an inverted phase contrast microscope (Nikon, Eclipse TS100) and captured with a stand-alone microscope camera controller (Nikon, DS-L3).

### Immunocytochemistry

HUVEC coverslips were visualized using immunocytochemistry. After removal from the systems, HUVECs were fixed using 4% paraformaldehyde (Fisher Scientific, cat #50-980-487) in 1 × PBS for 10 min at room temperature. Cells were then rinsed 3 times with 1 × PBS and permeabilized with a solution of 0.1% Triton X-100 (Sigma, Cat #X100-100ML) in 1 × PBS for 15 min at room temperature. Cells were then incubated for 1 h in a blocking solution of 5% goat serum in 1 × PBS at room temperature and then incubated with the primary antibodies in blocking solution overnight at 4 °C. The primary antibodies used were anti-CD31 [PECAM-1] (Thermo Scientific, cat #PA5-16301) and anti-CD54 [ICAM-1] (Thermo Scientific, cat #MA5407). The following day, cells were washed with 1 × PBS three times and following the third wash, cells were incubated with the secondary antibodies, which were conjugated to Alexa-488 (Thermo Scientific, cat #A-11001) or Alexa-568 (Thermo Scientific, cat #A-11011) fluorophores. This incubation was performed at room temperature for 2 h in the dark. Following incubation, cells were washed twice with 1 × PBS- and PBS was left on for 5 min with each wash. Cells were incubated with DAPI for 5 min followed by another series of two 5-min 1 × PBS washes. Coverslips were then mounted onto glass slides with an antifade mounting solution (Thermo Scientific, P36931).

### Cell viability

The viability of HUVECS and splenocytes was determined via the use of an Alamar blue solution (Thermo Scientific, DAL1025) to perform a metabolic reduction assay. After the removal of coverslips from the multi-organ systems, cells were incubated in 500 µL of 10% Alamar blue solution in 1 × PBS at 37 °C for 24 h. Following incubation, 100 µL were collected and transferred to a 96 well plate and read at a fluorescence excitation wavelength of 570 nm and emission at 590 nm using a BioTeK Synergy HT plate reader. The viability of the hepatocytes was determined via a standard MTT assay. MTT powder was dissolved in a common growth medium to a final concentration of 5 mg/mL. Cells were incubated in 500 µL of 5 mg/ml MTT powder in cell culture medium at 37 °C, 5% CO_2_. After 90 min, all medium was removed, and crystals were dissolved in 10% SDS with 0.5% acetic acid in DMSO. 100 µL of the solution was placed in a 96 well plate and absorbance read at 570 nm using a BioTek Synergy HT plate reader.

### High pressure liquid chromatography (HPLC) and mass spectroscopy (MS)

Drug concentrations were determined using an LCMS system consisting of a Thermo Scientific (Waltham, MA) Vanquish HPLC System interfaced to a Thermo Scientific Altis Triple Quadrupole Mass Spectrometer with an electrospray source. All drugs were separated using gradient elution methodology on a Biphenyl phase (Restek, 2.1 mmid, 100 mm length, 1.8 µm particle diameter) with binary mobile phase. Samples (50 µL) were diluted with methanol (200 µl) and centrifuged to precipitate proteins. The supernatant was diluted with a solution similar to the initial mobile phase that contained the internal standard, cisapride. The MS was set to positive ion mode and Selective Reactive Monitoring (SRM) with argon used for collision induced dissociation. The initial mobile phase was 20% methanol. Immediately following injection, the organic phase concentration was increased linearly to reach 95% methanol at 8 min. Then the mobile phase was returned to the initial condition (20% methanol) and held for 2 min to condition the column for the next injection. Both aqueous and organic phases contained 0.1% formic acid. SRM (parent/daughter) transitions used were m/z 320.19 → 247.125 for chloroquine and 466.12 → 184.042 for cisapride. Quantitation was based on a 7-point calibration curve.

### Statistical methods

Values are expressed as the mean ± standard error of the mean (SEM) of a minimum of three independent experiments. Data points were collected such that significant differences were able to be observed in the data, N = 3. Organ viability data was evaluated via one-way ANOVA followed by Dunnett’s test. For the determination of drug efficacy in the treatment sensitive and resistant models, parasitemia was normalized to the control and two-way ANOVA with Fisher’s LSD was conducted. Comparisons of parasitemia levels were done at each timepoint under the assumption that a transition from statistical significance to insignificance indicates recrudescence in that culture.

## Supplementary Information

Supplementary Information.

## Data Availability

All data generated or analyzed during this study are included in this published article (and its Supplementary Information files).
