# Supplementary material for: Development of a human malaria-on-a-chip disease model for drug efficacy and off-target toxicity evaluation
Source: Sci Rep. 2023 Jun 28;13:10509. doi: 10.1038/s41598-023-35694-4 (PMC10307889; doi:10.1038/s41598-023-35694-4)
Supplement: Supplementary file 1 — Supplementary Information. [file 41598_2023_35694_MOESM1_ESM.pdf]

Development of a Human Malaria-on-a-Chip Disease Model for Drug Efficacy and Off-Target Toxicity Evaluation

Michael J. Rupar, Trevor Sasserath, Ethan Smith, Brandon Comiter, Narasimhan Sriram, Christopher J Long, Christopher W McAleer, James J Hickman*

Supplementary Information

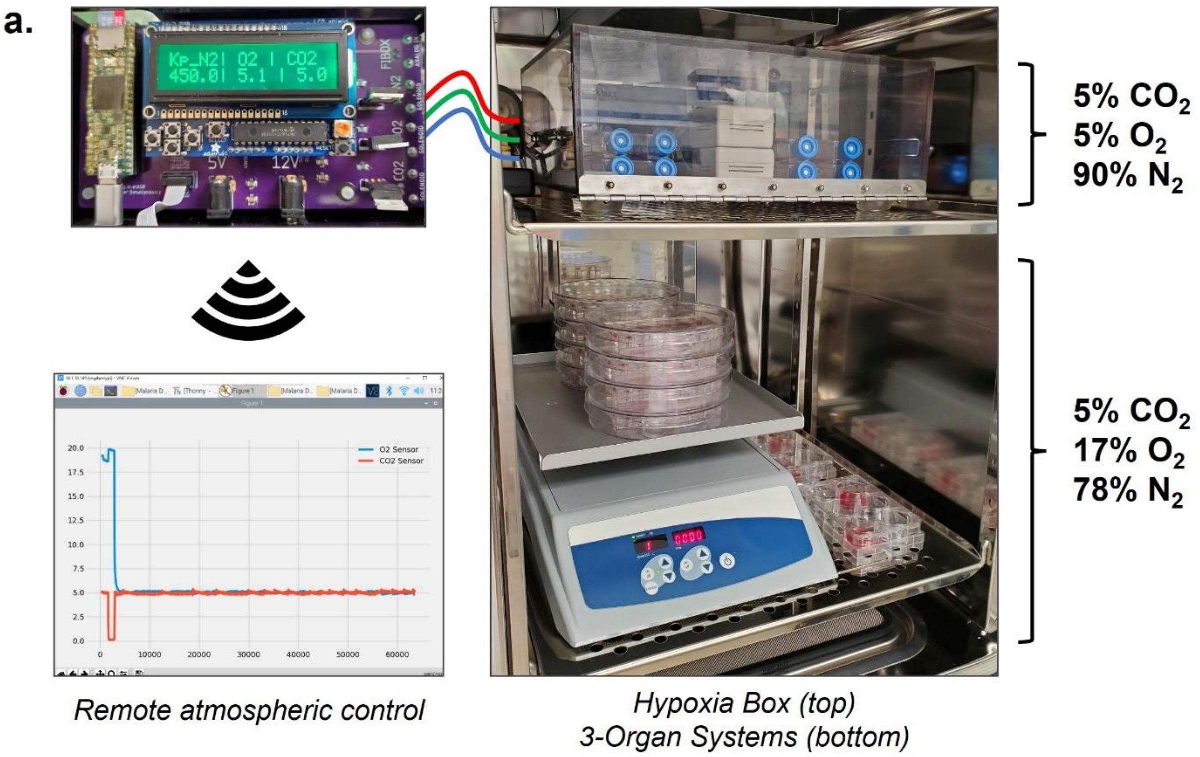

b.

Medium Component	Catalog Number	RPMI Complete
RPMI 1640 (+L-glut, +HEPES)	ThermoFisher 23400021	*Base*
Sodium Bicarbonate ($NaHCO_3$)	Sigma-Aldrich 36486	2.1 mg/mL
Albumax	ThermoFisher 11020021	4 mg/mL
Hypoxanthine	Sigma-Aldrich H9377	25 µg/mL
Pen-Strep	Sigma-Aldrich P4333	200 U/mL

Supplemental Figure 1. Hesperos hypoxia platform for long-term *P. falciparum* culture. (a) We have developed an internal environmental control unit for the continuous expansion of *P. falciparum* that is designed to fit in a standard CO_2 incubator. Atmospheric conditions inside the platform achieve desired gas concentrations within 30 minutes and can be monitored and adjusted remotely. (b) Medium formulation for expansion of *P. falciparum.*

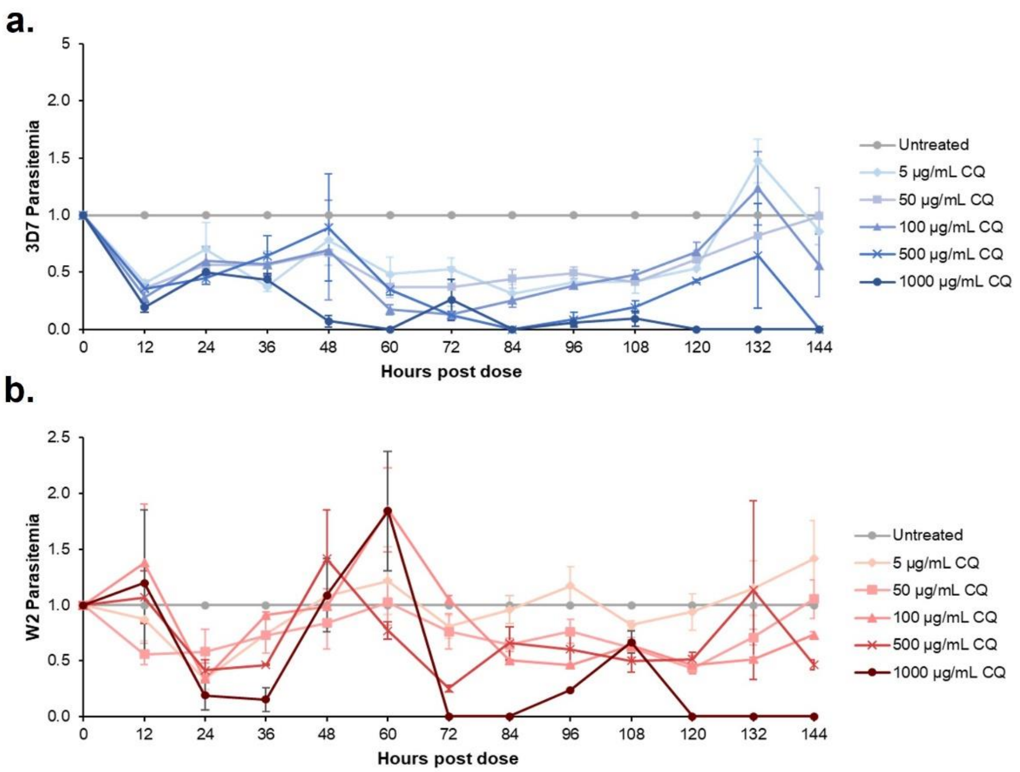

Supplemental Figure 2. Dose response chloroquine dosing in single-organ systems. (a) Effects of chloroquine dosing on systems infected with chloroquine sensitive 3D7 strain over 6 days. (b) Effects of chloroquine dosing on systems infected with chloroquine resistant W2 strain over 6 days.

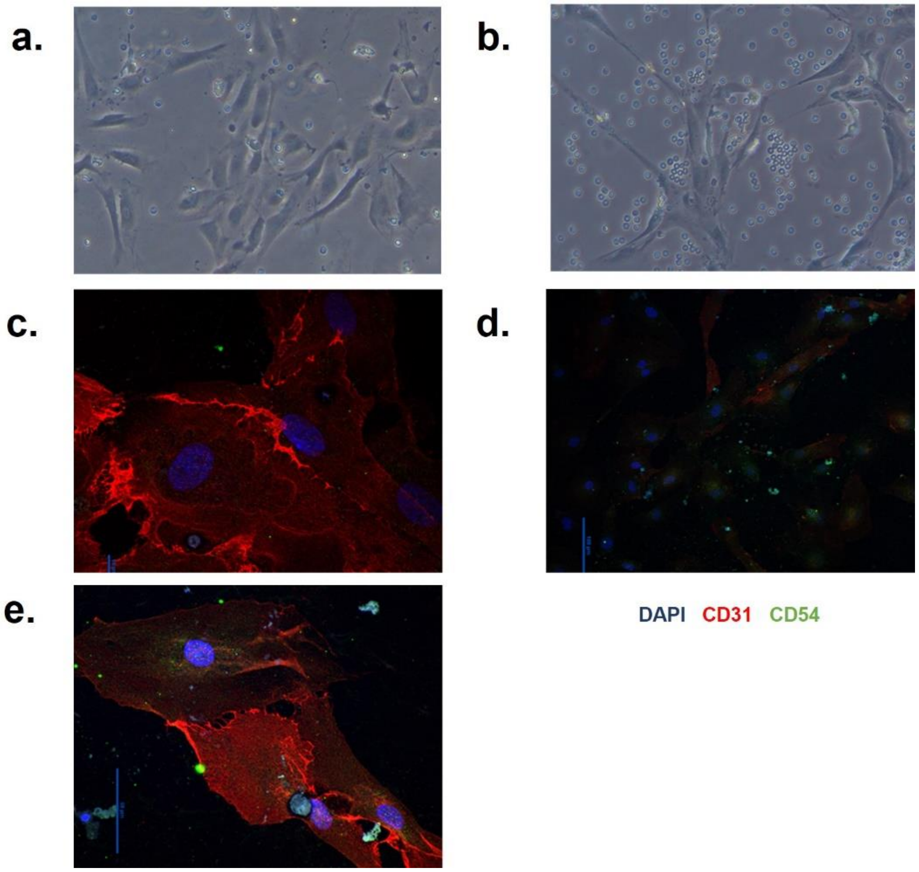

Supplemental Figure 3. Imaging of HUVECs from malaria-on-a-chip systems (a) Phase imaging of uninfected control system on day 8 following disassembly under 10X objective. (b) Phase imaging of system infected with *P. falciparum* on day 8 following disassembly under a 20X objective. Cells were washed twice with 1xPBS while gently agitating to remove any unadhered RBCs. Coverslips were fixed with cold 1% PFA for 30 minutes followed by 2 more washed with 1x PBS (c) ICC imaging of HUVECS from uninfected control systems. (d) ICC imaging of HUVECS from non-dosed *P. falciparum* infected systems. (E) ICC imaging of HUVECS from *P. falciparum* infected systems in the high chloroquine dose group.

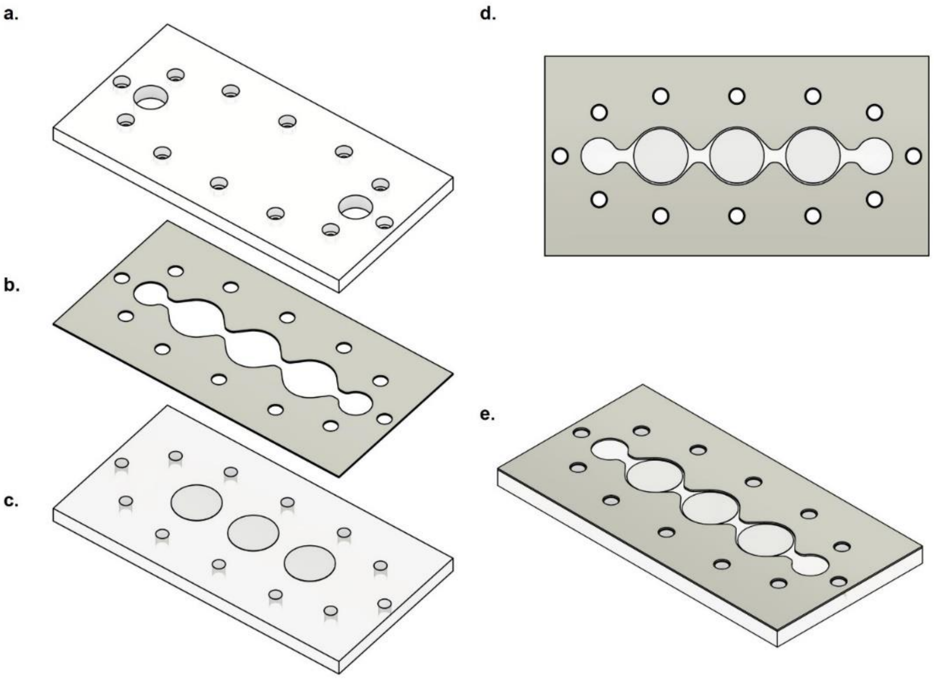

Supplemental Figure 4. For the malaria-on-a-chip system design consisted of an (a) acrylic top, (b) PDMS gaskets, and (c) an acrylic bottom. Coverslips were laid on the bottom housing, media added atop the coverslips, and 5/16” screws were then used to assemble the housing components together with the PDMS gaskets sandwiched between the top and bottom pieces. (d) Birds eye view of the Malaria-on-a-chip system and (e) an oblique view.
